# A Retrospective Cohort Study of Total Colonic Aganglionosis: Is the Appendix a Reliable Diagnostic Tool?

**DOI:** 10.21699/jns.v5i4.460

**Published:** 2016-10-10

**Authors:** T O'Hare, M McDermott, M O'Sullivan, P Dicker, B Antao

**Affiliations:** 1 Department of Paediatric Surgery, Our Lady's Children's Hospital, Dublin, Ireland; 2 Department of Histopathology, Our Lady's Children's Hospital, Dublin, Ireland; 3 School of Postgraduate Studies, Faculty of Medicine and Health Sciences, Royal College of Surgeons in Ireland, Dublin, Ireland

**Keywords:** Total colonic aganglionosis, Hirschsprung's disease, Appendix, Histology, Rectal suction biopsy

## Abstract

Background: Hirschsprung's disease (HD) is characterized by a lack of ganglion cells in the myenteric and submucosal plexus, associated with increased numbers of acetyl cholinesterase (AChE) positive nerve fibres. In approximately 10% of patients with HD the entire colon will be affected; a condition known as Total Colonic Aganglionosis (TCA). Aganglionosis of the appendix has long been considered to be an important finding in a patient in whom TCA is suspected, but its reliability for diagnosis has seldom been discussed. The aim of our study was to assess the reliability of the appendix as a histological specimen for the diagnosis of TCA, and to evaluate the long-term outcome of TCA.

Methods: A retrospective cohort study was performed of all pathological specimens of patients with confirmed HD in our institution between March 2006 and April 2016.

Results: Out of a total of 91 patients identified, 15 patients also had histopathological analysis of the appendix. Nine of these cases were confirmed as having TCA. The remaining 6 patients had HD involving the rest of the bowel up to the ascending colon, with normal ganglion present in the caecum. The appendix was removed in all the 15 cases. All 9 patients with confirmed TCA had aganglionosis of the appendix as well. The remaining 6 cases of short and long segment HD not involving the caecum, demonstrated normal ganglion cells within the appendix.

Conclusion: Aganglionosis of the appendix is a reliable tool in the diagnosis of TCA. The authors recommend that at the time of levelling biopsies, if aganglionosis extends to the mid-transverse colon, an ileostomy be performed and appendix sent for definitive confirmation of TCA. However, at the time of definitive surgery, a frozen section of pull-through segment of bowel is recommended to confirm the presence of ganglion cells.

## INTRODUCTION

TCA is a rare condition occurring in 5-15% of cases of HD and approximately in 1/50,000 lives births [1-3]. The traditional pathologists dictum has been that is difficult to identify myenteric plexus nerve cells in the appendix and therefore the appendix cannot be taken as representative of the rest of the small bowel and colon [4]. Despite the fact that aganglionosis of the appendix has long been considered an important finding in a patient in whom TCA is suspected, its reliability for diagnosis has seldom been discussed, with the majority of published data being on isolated case reports. The aim of our study was to evaluate the correlation between aganglionosis of the appendix and TCA, and to evaluate the long-term outcome of TCA.

## MATERIALS AND METHODS

This is a retrospective cohort study of all pathological specimens of patients with confirmed HD in our institution between March 2006 and April 2016. Demographic and clinical data of all cases of TCA was also collected and analysed. Institutional board ethical approval was obtained prior to commencement of this study.

Primary outcome analysis was the reliability of the appendix as a useful histological tool in TCA. Secondary outcome analysis was long term outcome of TCA.

A systematic literature search and review was also performed to identify articles of interest with respect to histological analysis of the appendix in HD. MEDLINE®/PubMed® and EMBASE® were searched using a combination of the terms "Hirschsprung's disease", "Aganglionosis", "Total Colonic Aganglionosis" and "Appendix". This returned a search result of 36 articles. After assessment, 5 of these articles were felt to be relevant to the topic [5-9]. A comparative analysis was done with our study and the other published relevant studies.

## RESULTS

A total of 91 patients were treated for HD and TCA between March 2006 and April 2016. Of these, 68 were male (75%) and 23 were female (25%); M:F was of 3:1. Sixty-nine patients had disease confined to the rectum or sigmoid colon (75%), seven had disease extending to the descending colon (8%), two had disease in the transverse colon (2%) and four had disease in ascending colon not extending to the caecum (4%). Overall 82 of the 91 patients had either short segment HD or long segment HD, not involving the entire colon (90%). The remaining 9 patients had TCA (10%). All 91 patients had their initial diagnosis made by rectal suction biopsy (RSBx) and confirmed by intraoperative levelling biopsy. Of these patients 76 of 91 (84%) were diagnosed within the first month of life, 86 of the 91 patients had been diagnosed by the second month (95%) and 90 of the 91 patients had been diagnosed by the third month of life (99%). Only a single patient was older than 3 months at diagnosis and this patient was a non-EU national. The mean age of diagnosis was 13.6 days (range; 3 to 115 days). 

Of the 91 patients who were treated for HD and TCA, 15 patients had histopathological analysis of the appendix (16%). Six of these patients were identified as having HD (40%) involving the ascending, transverse or rectosigmoid colon, with normal ganglion cells present in the caecum. The remaining 9 patients were identified as having TCA (60%) with aganglionosis seen to extend into the ileum. The 6 patients with HD and normal ganglion cells present in the caecum also had clearly demonstrated normal ganglion within the appendix. All 9 patients with confirmed TCA also had aganglionosis of the appendix (Fig. 1). The specificity and sensitivity were 100% for appendix as a diagnostic tool for TCA, in our study.

**Figure:**
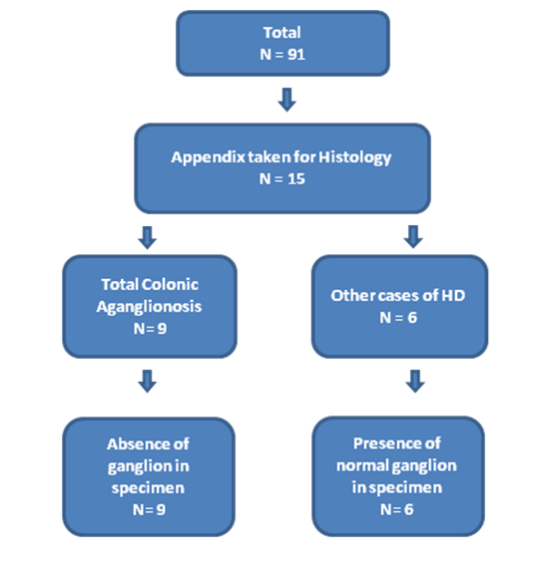
Figure 1: Flow chart outlining the distribution of all cases of Hirschsprung's disease and histological analysis of appendix.

Associated anomalies were present in 4 of the 9 patients (44%) with TCA (Table 1). All TCA patients had an initial defunctioning (first-stage) enterostomy performed at a mean age of 45 days (range: 5 to 144 days). All cases of TCA underwent a definitive procedure at a mean age of 388 days (range; 48 to 1125 days) [Table 2]. Four children underwent surgery within the first year of life, four within second year of life and one child after 3 year of age. Five patients (55%) had their ileostomy reversed at a mean age of 605 days (range; 43 to 1104 days). Post-operative complications were seen in 6 (66%) patients [Table 3]. The remaining three patients (33%) are currently well, with no ongoing issues at a mean follow-up period of 5.4 years (range: 1.5 to 7 years).

**Figure:**
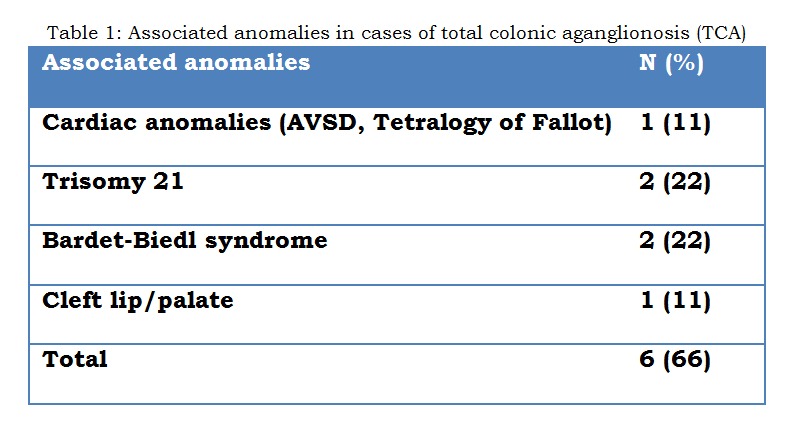
Table 1: Associated anomalies in cases of total colonic aganglionosis (TCA)

**Figure:**
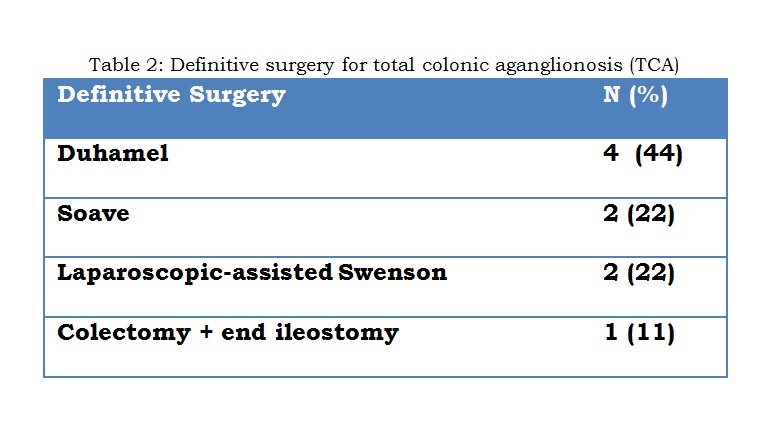
Table 2: Definitive surgery for total colonic aganglionosis (TCA)

**Figure:**
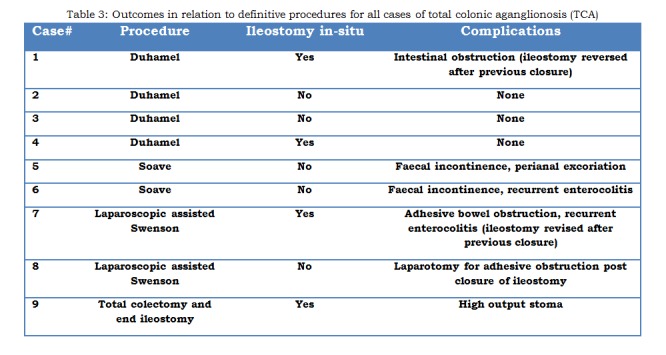
Table 3: Outcomes in relation to definitive procedures for all cases of total colonic aganglionosis (TCA)

## DISCUSSION

The concern while using appendix as a diagnostic tool in TCA is based on Kamoshita's finding in 1968, who described difficulty in identifying myenteric plexus nerve cells in the appendix, as the muscle wall is thin and the nerves are small [4]. Despite our small sample size, we found that demographics, comorbidities and outcomes in our study are generally similar to previously published data, with cases of TCA, having a less favourable outcome [10,11]. Our results refute the notion that the appendix is unreliable as an indicator of TCA. We achieved sensitivity and specificity of 100% when using the appendix as a marker of TCA. On reviewing the literature, N-Fekete et al [5], Anderson et al [6] and Shaw et al [7] had all previously remarked that in cases of TCA, the appendix invariably was aganglionic; however the investigation of this was not the primary aim of any of these studies. In contrast to these findings Lane et al [8] and Shih et al [9] have more recently published limited case reports wherein it was argued that an aganglionic appendix is not a reliable indicator of TCA.

In 1986, N-Fekete et al reviewed all cases of TCA at their institution between 1960 and 1984. They reported that in 17 cases of TCA where the appendix was available for histopathological analysis, the appendices were invariably aganglionic [5]. Likewise, both Anderson et al and Shaw et al, reported no significant difference in the ganglion cells of rectosigmoid HD, long segment HD and normal controls, while the appendix was invariably aganglionic in cases of TCA [6,7]. In contrast, Lane et al described a case of a newborn male infant with delayed passage of meconium, wherein a contrast enema failed to identify a transition zone and proceeded to diagnostic laparoscopy. The appendix was sent for histopathological analysis and was found to be aganglionic. The surgeons felt that this was in keeping with TCA and an ileostomy was fashioned. A later rectal suction biopsy revealed normal ganglion cells [8]. Similarly in 1998 Shih et al, described a case of a newborn infant who presented with acute onset perforation of the distal ileum. At laparotomy the appendix was sent for histopathological analysis and was found to be aganglionic and an ileostomy was fashioned. At re-exploration 7 months later ascending, transverse and sigmoid colon biopsies revealed the presence of normal ganglion cells [9]. It is difficult to ascertain the reason for the finding of an aganglionic appendix in an otherwise ganglionic bowel. There are 3 theoretical but plausible explanations. The first is misinterpretation of the histological sample. Secondly it has previously been described by Singh et al. that the ganglion cells in an acutely inflamed appendix are significantly altered [12]. This would certainly warrant considering in the case reported by Shih et al, as given the presence of an ileal perforation it is reasonable to assume that there may have been significant extrinsic inflammation of the appendix, which could have affected the histological analysis of the appendix. Finally while rare there are documented cases of zonal aganglionosis or skip-segment HD, whereby an area of aganglionosis is surrounded proximally and distally by ganglionic bowel [13,14]. This could explain the presence of an aganglionic appendix in an otherwise normally innervated colon.

## Conclusion

Based on our findings we advocate the following treatment protocol. Once the initial diagnosis of HD has been confirmed by rectal suction biopsy, stepwise distal to proximal levelling biopsies should be taken as usual. If aganglionosis extends to the mid transverse colon it is appropriate to send the appendix (assuming no other sources of intraabdominal sepsis) for histopathological analysis. If the appendix is aganglionic then a diagnosis of TCA can confidently be made and an ileostomy should be fashioned at least 10-15cm proximal to the ileocaecal valve. The ultimate choice of definitive surgery will be dependent on surgeon and centre familiarity and expertise. Finally at time of definitive surgery it is essential to perform levelling ileal biopsies as HD can extend into the ileum. 

## Footnotes

**Source of Support:** None

**Conflict of Interest:** None
